# Examining the Association Between Internet Addiction and Nonsuicidal Self-Injury Among Chinese Middle School Students: Prospective Cohort Study

**DOI:** 10.2196/86427

**Published:** 2026-06-17

**Authors:** Tianqing Fan, Lintong Song, Sihong Li, Xingyue Jing, Mireille Twayigira, Chunxiang Huang, Xuerong Luo, Yanmei Shen

**Affiliations:** 1 Department of Psychiatry, National Clinical Research Center for Mental Disorders, and National Center for Mental Disorders, The Second Xiangya Hospital of Central South University, No. 139, Renmin Middle Road, Furong District, Changsha, Hunan, 410011, China, 86 15116269263

**Keywords:** internet addiction disorder, self-injurious behavior, adolescents, sex characteristics, longitudinal studies

## Abstract

**Background:**

While cross-sectional studies have consistently reported an association between nonsuicidal self-injury (NSSI) and internet addiction (IA), longitudinal evidence regarding the directionality and dose-response relationship remains limited. Furthermore, the roles of sex and varying degrees of problematic internet use in predicting new-onset NSSI are not fully understood.

**Objective:**

This prospective cohort study aimed to investigate whether baseline IA and its intermediate states predict the subsequent new onset of NSSI among Chinese adolescents over a 6-month period and to explore potential sex differences in this longitudinal association.

**Methods:**

A prospective cohort design was used. A total of 1315 junior high school students without a history of NSSI were recruited at baseline, and 704 (53.5%) students completed the 6-month follow-up. IA and NSSI were assessed using the Chinese Internet Addiction Scale-Revised and a self-report questionnaire from the Adolescent Health-Related Risky Behavior Inventory, respectively. Logistic regression analysis was conducted to examine the predictive value of IA exposure for incident NSSI, adjusting for key covariates, including sex, age, ethnicity, only child status, anxiety, and depression. Restricted cubic spline regression was used to model the dose-response relationship between distinct states of IA and NSSI risk.

**Results:**

The baseline prevalence of IA was 9.09% (64/704). At the 6-month follow-up, the incidence rate of NSSI was 9.8% (69/704). Restricted cubic spline regression revealed a linear dose-response relationship, where the risk of incident NSSI escalated with increasing IA severity. In the fully adjusted model for the total sample, baseline IA was a significant predictor of subsequent NSSI (odds ratio [OR] 2.185, 95% CI 1.031-4.627; *P*=.04). Crucially, stratified analyses revealed significant sex disparities: the longitudinal association between IA and subsequent NSSI was statistically significant among female adolescents (OR 3.271, 95% CI 1.101-9.717; *P*=.03) and the intermediate internet-dependent state (OR 2.593, 95% CI 1.002-6.710; *P*=.049) but not among male adolescents (IA: *P*=.44; internet-dependent state: *P*=.87).

**Conclusions:**

NSSI incidence is notably prevalent among Chinese junior high school students. While IA serves as a robust, independent risk factor for predicting the new onset of NSSI in the overall adolescent population, sex-stratified analyses revealed that this longitudinal association was statistically significant (*P*=.03) only among female students. These findings underscore the critical need to integrate IA assessments into school-based mental health screenings and highlight the necessity of developing sex-specific, emotion-focused prevention strategies to mitigate NSSI risk.

## Introduction

Nonsuicidal self-injury (NSSI) is defined as committing direct and intentional self-harm behaviors without suicidal intent and frequently involves cutting or carving the skin [1]. It is quite common among young people, with a lifetime prevalence ranging from 17% to 60% [2]. It is significantly associated with higher risks of suicide and subsequent repeated NSSI [3]. The more frequent the NSSI, the higher the risk of suicidal behavior [4]. NSSI in adolescence is a strong indicator of mental health problems later in life [5] and is often comorbid with mental disorders, such as emotional disorders (depression [6], bipolar disorder [7], and others), neurodevelopmental disorders [8], and personality disorders [9]. There has been burgeoning interest in exploring the correlation between internet addiction (IA) and NSSI.

IA, also called problematic internet use, is characterized by excessive and inappropriate human-computer interaction behavior and is considered a behavioral addiction [10]. IA can serve as a coping mechanism for emotional distress, as described by the compensatory internet use theory, which posits that adolescents engage in excessive internet use as a maladaptive strategy to alleviate negative affect or escape real-life difficulties [11]. It might also be associated with serious consequences including suicidal behavior and emotional problems such as depression and anxiety [12], particularly in relation to NSSI in Chinese adolescents [13]. Individuals with IA may isolate themselves from social interactions, escape reality, have a desire for instant satisfaction, and experience challenges in managing negative emotions [14].

NSSI also functions as a maladaptive emotional coping mechanism [15], and reliance on the virtual world for emotional regulation is often insufficient or temporary. According to the experiential avoidance model [16], when low-intensity avoidance strategies, such as internet use, fail to dampen overwhelming distress or mask underlying symptoms of anxiety and depression, adolescents may retreat into the virtual world to temporarily alleviate negative affect. This may obscure underlying symptoms of anxiety or depression that would otherwise trigger clinical attention. When online activities fail to effectively regulate intensifying emotional pain, adolescents may resort to NSSI as a more drastic mechanism for emotional release [17]. This mechanism may involve a desire to avoid or escape distressing situations, as well as difficulties in regulating emotions. Additionally, potential psychological factors, such as impulsivity, low self-esteem, depression, or anxiety, may also contribute to NSSI [18]. Given the abundance of similarities, IA and NSSI may share underlying mechanisms. Theoretically, IA may serve as a maladaptive coping strategy that exacerbates emotional dysregulation and social isolation, thereby increasing vulnerability to NSSI through psychological pathways, such as diminished self-esteem and identity confusion. This theoretical link is supported by emerging longitudinal evidence in related mental health domains. For instance, large-scale prospective studies among Chinese adolescents have demonstrated that internet gaming disorder significantly predicts the incidence and persistence of suicidal ideation and depression over time [19,20]. Furthermore, a dose-response relationship has been observed between internet gaming disorder severity and the risk of first-onset suicide attempts [21]. However, despite these advances in understanding suicidality, specific longitudinal research on IA and NSSI remains limited. Most existing studies have explored this association cross-sectionally. Consequently, strictly longitudinal designs are urgently needed to clarify the directionality and determine whether IA represents a modifiable risk factor for early intervention in NSSI.

Given that previous studies have indicated sex differences in IA [22] and NSSI [23], this pathway may be moderated by sex due to distinct patterns of online behavior and coping mechanisms. Studies have indicated that male individuals with IA exhibit a higher susceptibility to gaming addiction, while female individuals with IA are more prone to challenges related to social networking [24], where upward social comparison and cyberbullying serve as potent triggers for internalizing problems that may precipitate NSSI. Furthermore, women demonstrate a greater tendency to use NSSI as a means of coping with psychological stress and distress [25]. Despite these plausible mechanisms, literature specifically examining sex differences in the longitudinal relationship between IA and NSSI remains limited. This study aims to investigate the effect of baseline IA on incident NSSI through a prospective cohort design and to explore whether there are sex differences in this context. We hypothesized that baseline IA predicts subsequent NSSI and that this association varies between male and female adolescents.

## Methods

### Study Population and Data Collection

A baseline survey was conducted in November and December 2020 to gather data from an urban public middle school located in Changsha, Hunan Province, China. To ensure a systematic recruitment procedure, the research team collaborated with school administrators and head teachers. Prior to the formal survey, study information sheets and electronic informed consent forms were distributed to all targeted students and their legal guardians. Using a cluster sampling method, all classes from Grades 7 to 9 were included. Initially, 1785 students were invited. However, 94 (5.3%) students declined to participate, and 120 (6.7%) students had incomplete information, resulting in their exclusion from the study. The inclusion criterion was students aged 12 to 18 years with no serious physical illness who could comprehend and respond to the questionnaires. The exclusion criterion was students whose parents and themselves declined to participate or did not provide informed consent for participation. A total of 1571 valid questionnaires (n=834, 53.1% male students and n=737, 46.9% female students) were collected at baseline. First, to establish a prospective cohort for incidence analysis, 256 (16.3%) students with prevalent NSSI at baseline were excluded. The follow-up survey took place in May and June 2021. Among the remaining 1315 eligible participants, 611 (46.5%) were lost to follow-up, primarily because ninth-grade students graduated during the study period. Consequently, a total of 704 (53.5%) students completed the follow-up survey after 6 months. Thus, these 704 middle school students were included in the follow-up analysis. A priori power analysis using G*Power 3.1 (Heinrich Heine University Düsseldorf) indicated that a minimum sample size of 308 was required; therefore, we intentionally oversampled at baseline (N=1785) to ensure sufficient statistical power despite the expected attrition in this longitudinal study.

### Main Exposure Variables

The Chinese Internet Addiction Scale-Revised (CIAS-R) was used for IA assessment. It is a 26-item scale designed by Chen et al in 2003 [26] to evaluate IA. Bai and Fan [27] revised it to a 19-item scale in 2005 and verified its validity and reliability in mainland adolescents in China (Cronbach α=0.90). This study used the validated 19-item version. A Likert scale with a 4-point rating system was used, where each item was assessed on a scale ranging from 1 (extremely inconsistent with my experience) to 4 (very consistent with my experience). The cutoff score is 53; a score above 53 is regarded as having IA, while a score between 46 and 53 is defined as an internet-dependent state, and a score below 46 is defined as normal. Consequently, participants were classified into three categories based on the CIAS-R total score: (1) normal (controls): score <46; (2) internet-dependent state: score 46 to 53, representing an intermediate or at-risk state; and (3) IA: score >53. In this study, the scale showed high internal consistency (Cronbach *α*=0.969).

### Outcome Assessment

NSSI was measured at baseline using the question “In the past 12 months, have you intentionally hurt yourself, but not for the purpose of taking your own life?” from the Adolescent Health-Related Risky Behavior Inventory, with “hit yourself,” “banged your head against something hard,” “pulled your own hair,” “scratched yourself,” “pinched yourself,” “bit yourself,” “cut yourself,” “burned yourself,” and “other methods” listed as examples. This instrument has been widely validated and extensively used in epidemiological studies among Chinese adolescents, demonstrating satisfactory test-retest reliability and construct validity [28-30]. NSSI was assessed at the 6-month follow-up using the same question, but the period was altered to the last 6 months. Participants were requested to select a yes or no response, and if they selected yes, they were asked to specify the NSSI methods they used.

### Assessment of Covariates

Basic sociodemographic information, such as age, sex, only child status, and ethnicity, were collected. Depression and anxiety in the past week were collected by the 2 subscales of the Depression Anxiety Stress Scale (DASS-21) [31]. Each subscale includes 7 items, and each item is rated on a 4-point Likert scale ranging from 0 (extremely inconsistent with my experience) to 3 (very consistent with my experience). Higher scores indicate more severe emotional problems, and the cutoff scores for the presence of depression and anxiety were 5 and 4 (raw score), respectively, based on the original criteria. This scale has been commonly used in Chinese adolescents and has proven to have good reliability, validity, and high practical value [32]. In this study, the Cronbach α coefficients for depression and anxiety subscales were 0.888 and 0.841, respectively.

### Procedure

The longitudinal study was implemented in 2 waves. As described in the *Study Population and Data Collection* section, the baseline survey was conducted in November and December 2020, followed by a 6-month follow-up in May and June 2021. Both waves used an online survey distributed via WeChat (Tencent Holdings Ltd). To ensure data matching between baseline and follow-up while maintaining confidentiality, student ID numbers were used as unique identifiers. Before the survey, all the schoolteachers who participated in this study were trained uniformly on how to deliver the survey, how to guide the students to complete the questionnaire, and how to assist when needed. Notably, students completed the survey in classrooms under uniform supervision to ensure standardized conditions and independent completion.

### Ethical Considerations

The ethics committee of the Second Xiangya Hospital of Central South University thoroughly reviewed and approved the use of the data, ensuring compliance with ethical standards (approval (2019) Lun-Shen [Ke] 021). Additionally, prior informed consent was obtained from all participant students and their legal guardians. All the students and their caregivers were made aware that participation in the study was entirely voluntary and that they could opt out at any time. To safeguard privacy and confidentiality, all collected data were deidentified; student ID numbers were temporarily used solely for matching baseline and follow-up surveys, after which the dataset was fully anonymized. Finally, participants did not receive any financial compensation for their involvement in this study.

### Statistical Analysis

Chi-square tests were used to compare categorical variables. After excluding NSSI at baseline, the association between baseline IA and new-onset NSSI during a 6-month follow-up was examined by logistic regression analysis. We built 3 models for the data. Model 1 calculated the crude odds ratios (ORs) with a 95% CI without adjusting for any covariable; model 2 adjusted for age, sex, only child status, and ethnicity; and model 3 adjusted for age, sex, only child status, ethnicity, and emotional state (including anxiety and depression). Furthermore, sex differences were also explored by constructing models hierarchically by sex. Statistical analyses were conducted using SPSS (version 23.0; IBM Corp) for chi-square tests and logistic regression, while R software (version 4.3.1; R Foundation for Statistical Computing) was used for restricted cubic spline analysis. Significance was set at *P*<.05. The reporting of this study adheres to the STROBE (Strengthening the Reporting of Observational Studies in Epidemiology) guidelines for cohort studies (Checklist 1).

## Results

### Baseline Characteristics of the Final Analytical Sample

**Figure 1.**
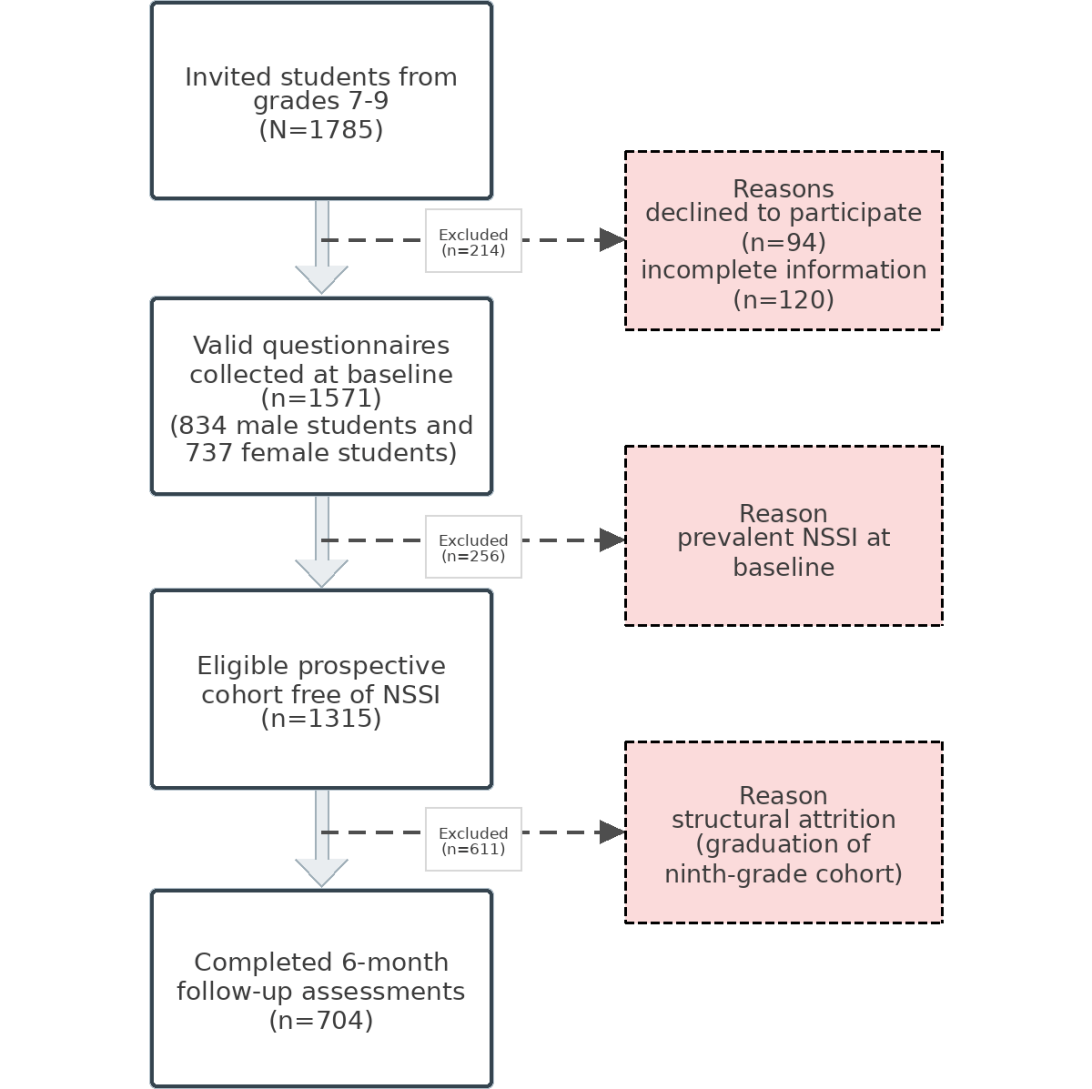
Flowchart of participant recruitment and 6-month follow-up in the prospective cohort study investigating incident nonsuicidal self-injury (NSSI) among middle school students (Changsha, China; November 2020 to June 2021).

A total of 704 participants who were free of NSSI at baseline completed the assessments and were included in the final analytical sample (Figure 1). The cohort comprised 395 (56.1%) male and 309 (43.9%) female participants, with an average age of 12.60 (SD 0.61) years. Based on the CIAS-R criteria, the baseline prevalence of the internet-dependent state was 11.7% (n=82), and the prevalence of IA was 9.1% (n=64). Baseline demographic comparisons revealed no significant intergroup differences among the normal, internet-dependent state, and IA groups concerning age, sex, or only child status (all *P*>.05); a statistically significant difference was observed regarding ethnicity, primarily driven by the overwhelming majority of Han participants, which is representative of the regional demographic profile. However, regarding clinical characteristics, students in the internet-dependent state and IA groups exhibited a significantly higher baseline prevalence of depression (*P*<.001) and anxiety (*P<.*001) compared with the normal control group. The consolidated demographic and clinical characteristics of the final analytical sample are summarized in Table 1. During the 6-month follow-up period, the overall incidence rate of new-onset NSSI among the final analytical cohort was 9.8% (69/704). To assess potential attrition bias, baseline characteristics were compared between participants who completed the follow-up and those who were lost. Results showed no significant differences in sex or baseline IA scores (all *P*>.05), indicating no systematic bias related to the primary exposure variable. Although the attrition group was significantly older due to graduation (*P*<.001; refer to Multimedia Appendix 1 for details), age was included as a covariate in all subsequent multivariate logistic regression models to statistically adjust for this baseline discrepancy.

**Table 1.** Baseline demographic characteristics of the study population between groups with different internet addiction (IA) status in a prospective cohort of junior high school students (Changsha, China; November to December 2020).

		Normal (n=558)	IDS^a^ (n=82)	IA (n=64)	*F* test/*χ*^2b^(*df*)	*P* value
Age (y), mean (SD)		12.60 (0.61)	12.63 (0.58)	12.53 (0.76)	0.5 (2,701)	.60
Sex, n (%)					2.8 (2)	.25
Male		306 (54.8)	47 (57.3)	42 (65.6)		
Female		252 (45.2)	35 (42.7)	22 (34.4)		
Ethnicity, n (%)					11.5 (2)	.003
Han		528 (94.6)	81 (98.8)	55 (85.9)		
Other		30 (5.4)	1 (1.2)	9 (14.1)		
Only child status, n (%)					2.1 (2)	.26
Yes		233 (41.8)	32 (39.0)	20 (31.3)		
No		325 (58.2)	50 (61.0)	44 (68.8)		
Depression, n (%)					49.5 (2)	<.001
No		437 (78.3)	43 (52.4)	29 (45.3)		
Yes		121 (21.7)	39 (47.6)	35 (54.7)		
Anxiety, n (%)					36.8 (2)	<.001
No		347 (62.2)	29 (35.4)	21 (32.8)		
Yes		211 (37.8)	53 (64.6)	43 (67.2)		
Nonsuicidal self-injury, n (%)					9.9 (2)	.007
No		513 (91.9)	70 (85.4)	52 (81.3)		
Yes		45 (8.1)	12 (14.6)	12 (18.8)		

a IDS: internet-dependent state.

b *F* values were used for continuous variables, and chi-square values were used for categorical variables.

### Longitudinal Association and Dose-Response Relationship

**Figure 2.**
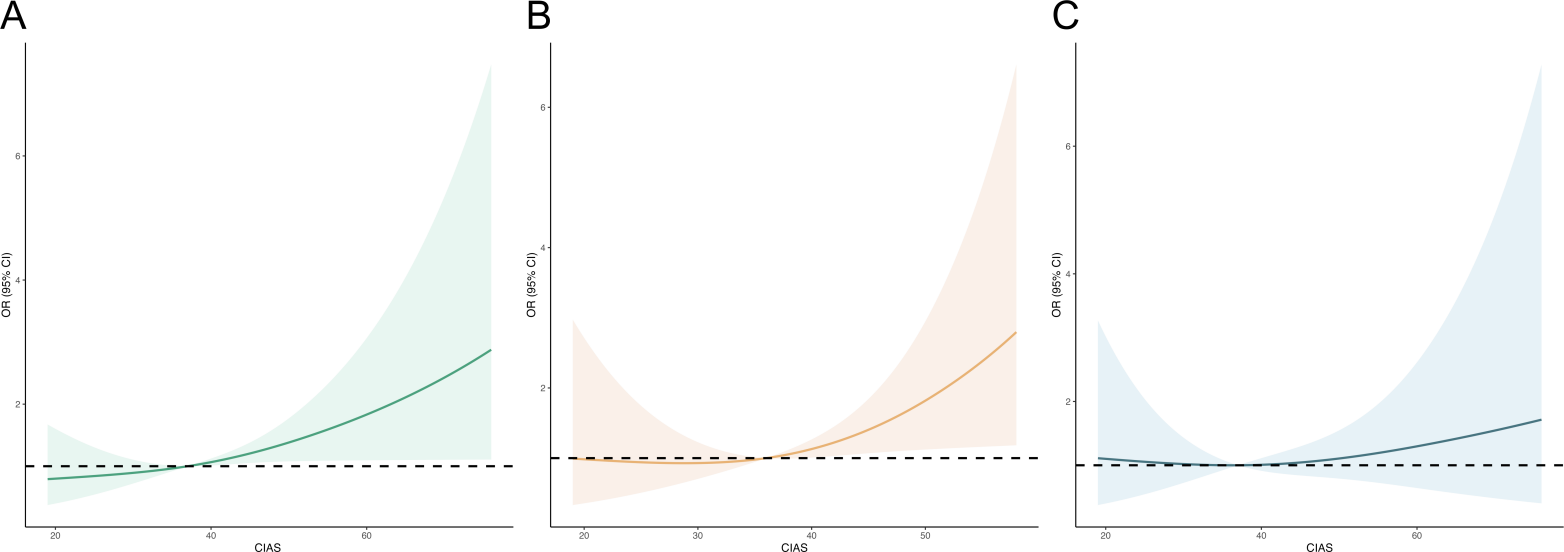
Restricted cubic spline regression analysis of the dose-response relationship between baseline internet addiction (IA) and the risk of incident nonsuicidal self-injury (NSSI) at 6-month follow-up in a prospective cohort of adolescents (Changsha, China; 2020‐2021). The x-axis represents scores from the Chinese Internet Addiction Scale (CIAS), while the y-axis represents the odds ratio (OR) from logistic regression for the risk of new occurrences of NSSI behavior 6 months later. (A) Model A denotes the entire participant population, (B) model B represents the female cohort, and (C) model C represents the male cohort. All models were adjusted for covariates, including age, ethnicity, anxiety, and depression. Model A was further adjusted for sex.

Significant ORs for NSSI were observed in IA (Table 2: models 1, 2, and 3). The crude OR of IA for NSSI was 2.631 (95% CI 1.309‐5.286; *P*=.007), and the significance remained after controlling for age, sex, ethnicity, and only child status in model 2, with an adjusted OR of 2.587 (95% CI 1.257‐5.321; *P*=.01). The association remained significant when further controlling for anxiety and depression in model 3, with an adjusted OR of 2.185 (95% CI 1.031‐4.627; *P*=.04). In contrast, no significant longitudinal association was observed between the internet-dependent state and subsequent NSSI. Specifically, even in the fully adjusted model (model 3), the internet-dependent state did not significantly predict incident NSSI (adjusted OR 1.810, 95% CI 0.881‐3.717; *P*=.11). These findings suggest that the elevated risk of incident NSSI is specifically driven by full IA rather than its intermediate state. Furthermore, in the restricted cubic spline regression models, the correlation between continuous IA severity and the risk of subsequent NSSI was displayed linearly (Figure 2).

**Table 2.** Longitudinal association between baseline internet addiction (IA) and the 6-month incidence of nonsuicidal self-injury (NSSI) among adolescents: odds ratios (ORs) and 95% CIs from a prospective cohort study (Changsha, China; 2020‐2021).

	NSSI, n (%)	Model 1^a^		Model 2^b^		Model 3^c^	
		OR (95% CI)^d^	*P* value	OR (95% CI)^e^	*P* value	OR (95% CI)^f^	*P* value
Normal (reference)	45 (8.1)	1.00	—^g^	1.00	—	1.00	—
IDS^h^	12 (14.6)	1.95 (0.99‐3.87)	.06	2.09 (1.04‐4.20)	.04	1.81 (0.88‐3.72)	.11
IA	12 (18.8)	2.63 (1.31‐5.29)	.007	2.59 (1.26‐5.32)	.01	2.19 (1.03‐4.63)	.04

a Model 1: crude OR.

b Model 2: OR adjusted for sex, age, ethnicity, and only child status.

c Model 3: OR adjusted for sex, age, ethnicity, only child status, anxiety, and depression

d *P* value for trend<.001.

e *P* value for trend=.009.

f *P* value for trend=.06.

g Not applicable.

h IDS: internet-dependent state.

### Sex-Stratified Analyses

**Figure 3.**
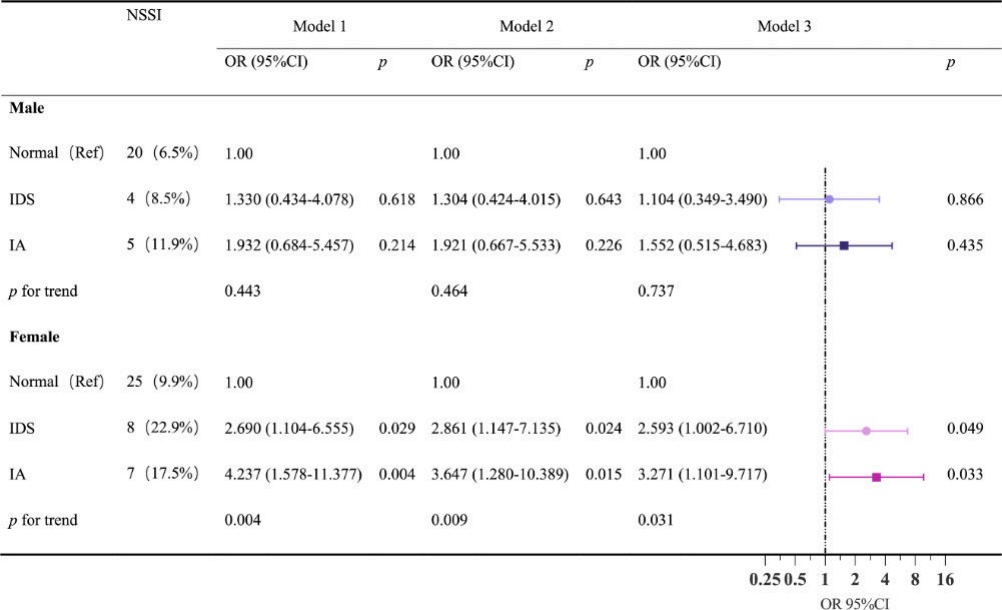
Sex-stratified analysis of the longitudinal association between baseline internet addiction (IA) and incident nonsuicidal self-injury (NSSI) at 6-month follow-up among a prospective cohort of adolescents (Changsha, China; 2020‐2021). Model 1: crude odds ratio (OR); model 2: OR adjusted for age, ethnicity, and only child status; and model 3: OR adjusted for age, ethnicity, only child status, anxiety, and depression. IDS: internet-dependent state.

The sex-based hierarchical model (Figure 3) did not reveal any significant OR for either the internet-dependent state or IA among male adolescents across all models (all *P*>.05). Conversely, for female adolescents, both the internet-dependent state and IA emerged as significant predictors of incident NSSI across all 3 models. Importantly, these results maintained their significance even after adjusting for age, ethnicity, only child status, anxiety, and depression (adjusted OR 3.271, 95% CI 1.101‐9.717; *P*=.03), and the adjusted OR for the internet-dependent state was also statistically significant (adjusted OR 2.593, 95% CI 1.002‐6.710; *P*=.049). Furthermore, it is noteworthy that in each of these models, the OR for IA surpassed that of the internet-dependent state.

## Discussion

This study is the first prospective cohort investigation to emphasize sex differences to examine the longitudinal association between IA exposure and the new onset of NSSI among Chinese adolescents. Our key findings are as follows: (1) baseline IA, but not the intermediate internet-dependent state, was a robust independent predictor of incident NSSI in the overall sample; (2) significant sex disparities were identified, with both the internet-dependent state and IA significantly associated with subsequent NSSI *only* among female adolescents, indicating a sex-specific dose-response relationship; and (3) the lack of overall internet-dependent state significance is likely due to a “sex masking” effect, highlighting the extreme vulnerability of female adolescents to even subclinical internet dependence. Together, these findings highlight the independent predictive value of IA for adolescent NSSI and underscore the critical need for sex-specific prevention strategies.

This study found that baseline IA might predict the risk of new-onset NSSI among adolescents, which is consistent with findings from a previous longitudinal design study [33]. This study used a multivariate logistic regression analysis with IA at baseline as a predictor of new self-harm or suicidal tendencies in the next year. Status classified as IA had a relative risk of new NSSI compared with those without, and this association remained significant after adjusting for several confounding variables (eg, baseline depression and anxiety levels). This suggests that while emotional distress is undoubtedly a contributor, IA appears to add a unique, incremental risk for subsequent NSSI behavior. Consistent with the experiential avoidance model and compensatory internet use theory discussed earlier, when the internet is used as an avoidant coping mechanism for emotional distress, its failure to adequately regulate intensifying negative emotions may precipitate NSSI as a more severe regulatory strategy [15]. It is worth noting that the internet-dependent state did not show a significant association with incident NSSI in the total sample across all models (all *P*>.05). This suggests a “clinical threshold” in the general adolescent population, where intermediate use is generally insufficient to precipitate NSSI, and only full IA acts as a robust independent predictor. However, significant associations were observed for the internet-dependent state across all models in the female subgroup. The overall nonsignificance likely resulted from a “sex masking” effect, concealing the extreme vulnerability of female adolescents. This suggests that the association of the internet-dependent state with NSSI may be highly sex specific. Mechanistically, because female adolescents are more prone to social networking, even moderate problematic use rapidly exposes them to upward social comparison, cyberbullying, and appearance anxiety, potent triggers for self-harm. In contrast, male adolescents predominantly use internet gaming for emotional venting, requiring a much higher addiction threshold (IA) to manifest NSSI. This highlights the critical importance of early sex-targeted monitoring when intervening with female adolescents in the at-risk internet-dependent state stage.

The sex stratification model indicated significant associations between IA and subsequent NSSI only in female adolescents with both an internet-dependent state and IA, which is inconsistent with previous findings. Surprisingly, only 2 cross-sectional studies have investigated the sex difference between IA and NSSI. A study of 11,356 adolescents in 11 European countries showed no significant sex interactions in IA and NSSI [34]. Another study enrolled 15,623 adolescents in China and showed that among adolescents aged 11 to 14 years, the ORs of IA for NSSI were greater in male adolescents (1.53, 95% CI 1.25‐1.88) than in female adolescents (1.13, 95% CI 0.90‐1.47) [13]. Various factors might contribute to sex differences, such as biological dissimilarities [35], differences in social status and expectations, variations in perceived social support [36], and varying susceptibilities to stress-related concerns [37]. The inconsistent results could be explained by the following aspects. First, variations in sex distribution among diverse participant samples and the complexity of defining IA may be associated with the final results. Second, the nature of internet use might play a pivotal role. Previous studies have unveiled sex-based IA disparities: male adolescents have a propensity for gaming addiction [38], which may serve as a more temporary distraction from distress rather than a direct source of negative self-evaluation, whereas female adolescents display increased vulnerability to social media–related problems [39], which fosters maladaptive social comparisons and cyberbullying victimization—both of which are potent triggers for body dissatisfaction and subsequent NSSI. Another study found that the sex differences in the association between different forms of internet use and NSSI were significant [40]. This study investigated the correlation between NSSI and IA with or without game play and found that nongamers had higher NSSI risk. The gamers were primarily male (27% female and 73% male), while the nongamers were predominantly female (78.8% female and 21.2% male). This finding may support a higher risk of NSSI in female adolescents with IA. This also suggests that we need to be more cautious in drawing conclusions when analyzing sex differences. Third, both IA and NSSI involve difficulties in emotional regulation and serve as a coping strategy [41,42], and IA in female adolescents is associated with an increased likelihood of emotional issues [43], and female adolescents are more likely to engage in rumination and internalizing coping styles when faced with online stressors. This pattern is strongly linked to both IA and NSSI, and these individuals may be more prone to adopting maladaptive coping strategies during episodes of emotional distress [44]. However, due to the current scarcity of research, further studies on sex differences are needed.

There are some limitations that need to be noted. First, the assessment of NSSI relied on a single binary item (yes or no), and while this measure was sufficient to identify the onset of the behavior in our longitudinal design**,** it constitutes a major limitation because it fails to assess its frequency, severity, methods, or functional motivations. Additionally, the data were based on retrospective self-report scales, which may introduce recall and social desirability biases. Future studies should use diagnostic interview tools to capture the multidimensional nature of NSSI and mitigate these measurement biases. Second, the attrition rate was relatively high (46.46%) due to the graduation of grade 9 students during the follow-up period. While this loss was structural, it may have excluded older adolescents from the final analysis and potentially introduced selection bias. Furthermore, the sample size for the subgroup of female adolescents with IA was relatively small (22/704, 3.1%). Although the association with incident NSSI was statistically significant, this limited sample size reduces statistical power and warrants caution in generalizing the sex-stratified findings. Third, although this study controlled for several confounding variables that might explain the association between IA and NSSI in adolescents such as depression and anxiety, it is noteworthy that other psychosocial variables such as family conflict and bullying victimization were not included. Future studies should include these measures to enhance understanding of the causal pathways.

In conclusion, our study reveals a significant association between IA and new-onset NSSI 6 months later, indicating that NSSI is prevalent in Chinese adolescents and that female adolescents with both IA and an internet-dependent state had a higher NSSI risk, which provides enhanced evidence for clinicians for the early identification and intervention of NSSI. These findings also emphasize the need for further research to better understand the mechanisms underlying this relationship between them. These findings have broader implications for public health policy, suggesting that school-based mental health screenings should integrate assessments of IA. Furthermore, intervention programs targeting NSSI prevention may benefit from sex-specific strategies that address IA as a modifiable risk factor.

## Supplementary material

10.2196/86427 Multimedia Appendix 1 Comparison of baseline characteristics between participants who completed the 6-month follow-up and those lost to follow-up.

10.2196/86427 Checklist 1 STROBE checklist.

## Abbreviations

CIAS-R: Chinese Internet Addiction Scale-Revised
DASS-21: Depression Anxiety Stress Scale
IA: internet addiction
NSSI: nonsuicidal self-injury
OR: odds ratio
STROBE: Strengthening the Reporting of Observational Studies in Epidemiology
