# Supplementary material for: Examining the Association Between Internet Addiction and Nonsuicidal Self-Injury Among Chinese Middle School Students: Prospective Cohort Study
Source: J Med Internet Res. 2026 Jun 17;28:e86427. doi: 10.2196/86427 (PMC13274967; doi:10.2196/86427)
Supplement: Multimedia Appendix 1 [file jmir-v28-e86427-s001.docx]

Table S1 Comparison of baseline characteristics between participants who completed the 6-month follow-up and those lost to follow-up.

|  |  | **COMPLETED**  N=704 | **LOST**  N=611 | F/χ^2^ | *p* |
| --- | --- | --- | --- | --- | --- |
| Age  Mean (SD) |  | 12.60(0.62) | 13.70(0.89) | 25.79 | ＜0.001 |
| CIAS  Mean (SD) |  | 36.72(13.36) | 37.91(14.90) | 1.537 | 0.062 |
| Gender (%) | Male | 395(56.1) | 341(55.8) | 0.012 | 0.914 |
|  | Female | 309(43.9) | 270(44.2) |  |  |
| Ethnicity (%) | Han | 664(94.3) | 581(95.1) | 0.387 | 0.534 |
|  | Other | 40(5.7) | 30(4.9) |  |  |
| Only child (%) | Yes | 285(40.5) | 253(41.1) | 0.116 | 0.734 |
|  | No | 419(59.5) | 385(58.6) |  |  |
| Depression (%) | No | 509(72.3) | 447(73.2) | 2.921 | 0.087 |
|  | Yes | 195(27.7) | 164(26.8) |  |  |
| Anxiety (%) | No | 397(56.4) | 373(61.0) | 2.921 | 0.087 |
|  | Yes | 307(43.6) | 238(39.0) |  |  |

Abbreviations: IA, Internet Addiction; IDS, Internet Dependent State.
